# Supplementary material for: Enhancing Coleoptile Length of Rice Seeds under Submergence through NAL11 Knockout
Source: Plants (Basel). 2024 Sep 17;13(18):2593. doi: 10.3390/plants13182593 (PMC11434697; doi:10.3390/plants13182593)
Supplement: Supplementary file 1 [file plants-13-02593-s001.zip › plants-3145583-supplementary-revised.pdf]

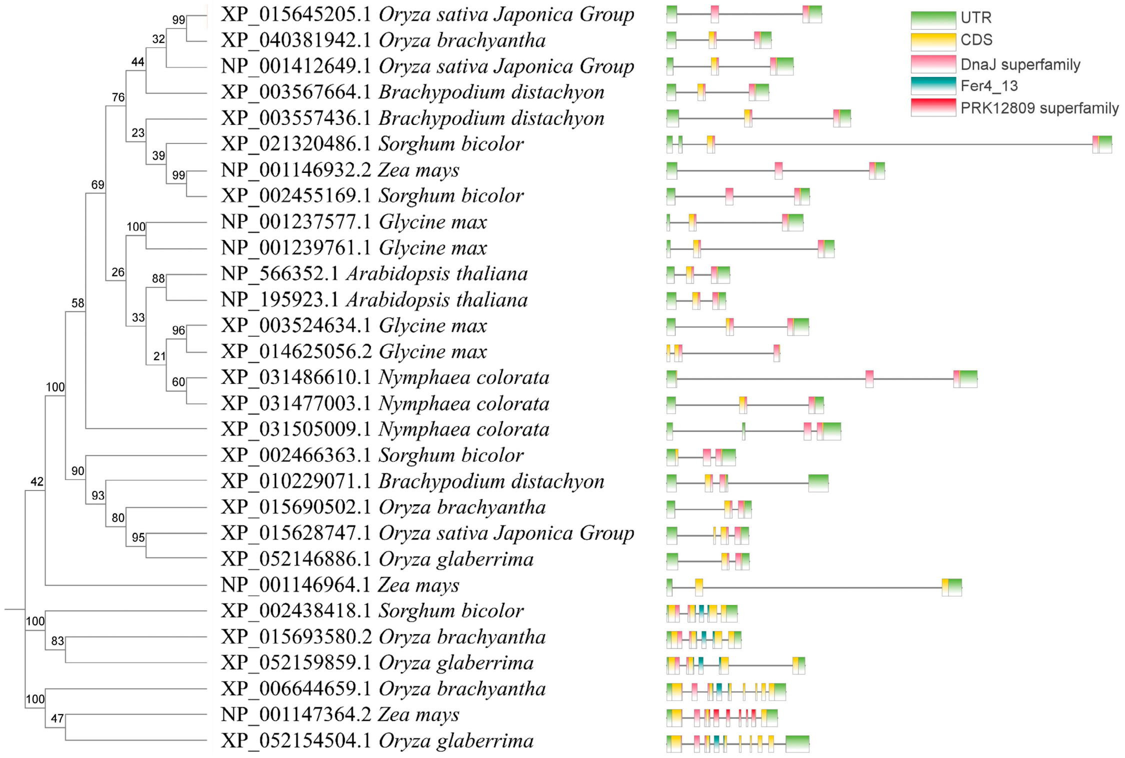

Fig. S1 Protein domain and gene structure analysis of *NAL11* and 28 homologous genes. Schematic diagram of *NAL11* proteins and 28 homologous proteins functional domain; Structural analysis of *NAL11* and 28 homologous genes based on their own genome annotation file GFF3; Exon－intron structure of the 28 homologous genes, with introns indicated by black lines, exons by yellow wedges, and upstream (5’)/downstream (3’) untranslated regions (UTRs) by green rectangles.

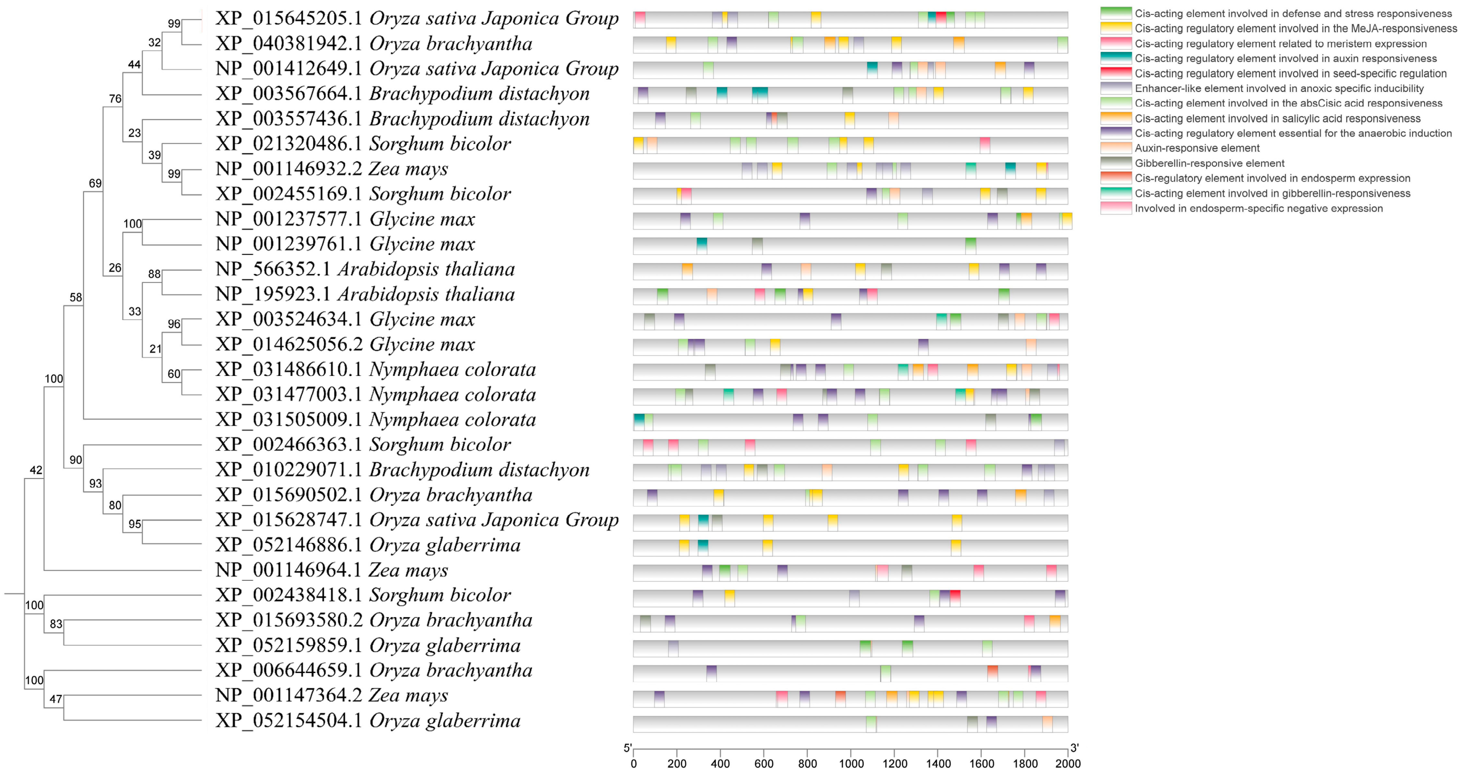

Fig. S2 Promoter prediction analysis of *NAL11* and 28 homologous genes, with introns indicated by black lines, exons by yellow wedges, and upstream (5')/downstream (3') untranslated regions (UTRs) by green rectangles. D, Distribution of various cis-elements in the promoters of 28 homologous genes. E, Color-coded patterns corresponding to each motif box.

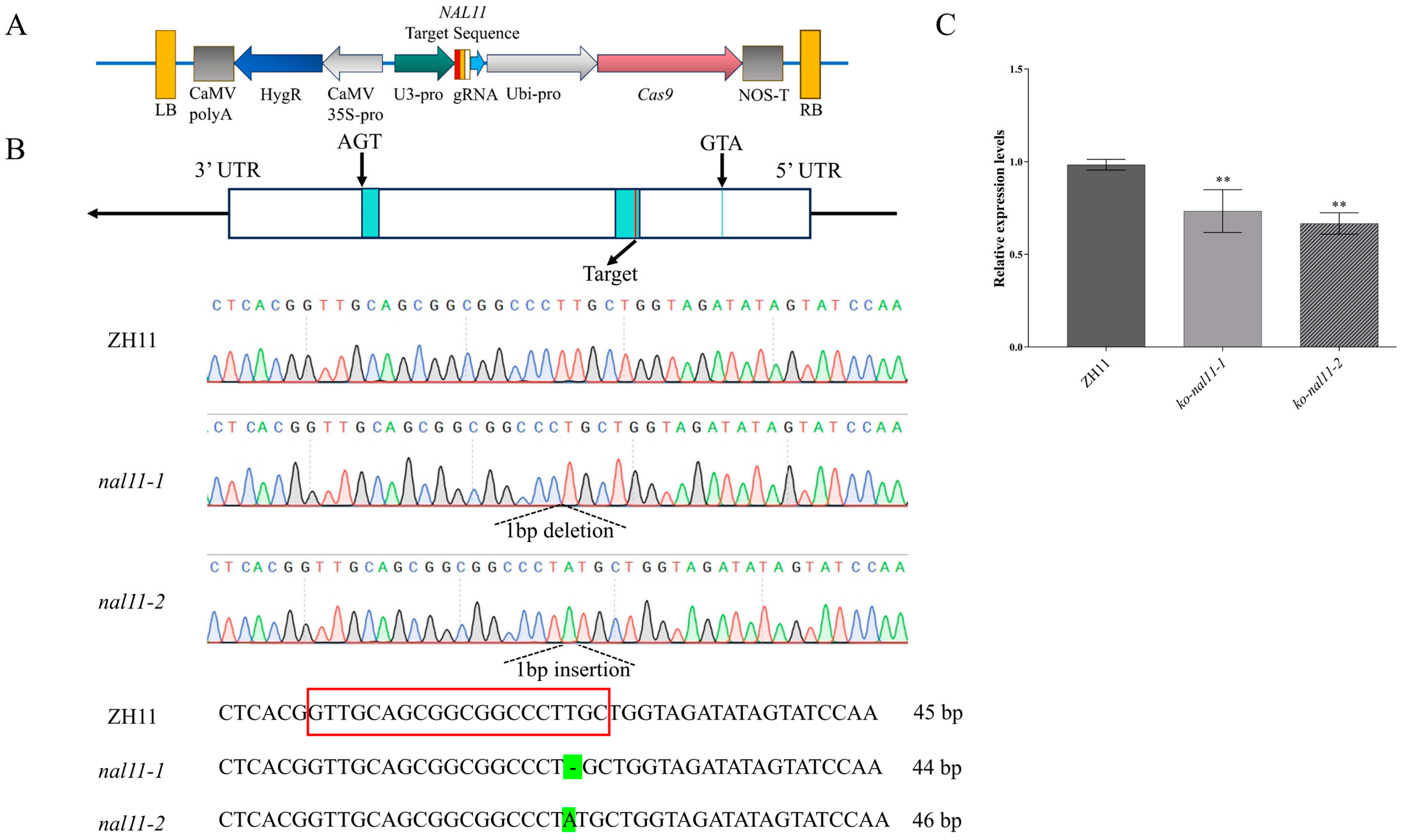
A
NAL11
Target Sequence
LB
CaMV
polyA
HygR
CaMV
35S-pro
U3-pro
gRNA
Ubi-pro
Cas9
NOS-T
RB
B
AGT
GTA
3’ UTR
5’ UTR
Target
ZH11
nal11-1
1bp deletion
nal11-2
1bp insertion
ZH11 CTCACGGTTGCAGCGGCGGCCCTTGCTGGTAGATATAGTATCCAA 45 bp
nal11-1 CTCACGGTTGCAGCGGCGGCCCT-GCTGGTAGATATAGTATCCAA 44 bp
nal11-2 CTCACGGTTGCAGCGGCGGCCCTATGCTGGTAGATATAGTATCCAA 46 bp
C
Relative expression levels
1.5
1.0
0.5
0.0
**
**
ZH11
ko-nal11-1
ko-nal11-2

Fig. S3 A, Schematic of the CRISPR/Cas9 knockout vector cloning of *NAL11*. B, Gene structure diagram of *NAL11*. The UTRs and CDS are indicated by black and orange rectangles, respectively; the black arrows indicate the start (ATG) and stop codon (TGA). The red box indicates the location of the target. The green background fonts indicate the mutation site of *NAL11* in the three mutants. Sequence length is shown below. C, Expression levels of *NAL11* in transgenic lines and wild type. (Data are presented as mean ± SD, $n = 3$; significant differences were determined by two-tailed Student's t-tests. $^{*}P < 0.05$, $^{**}P < 0.01$, ns, no significance).

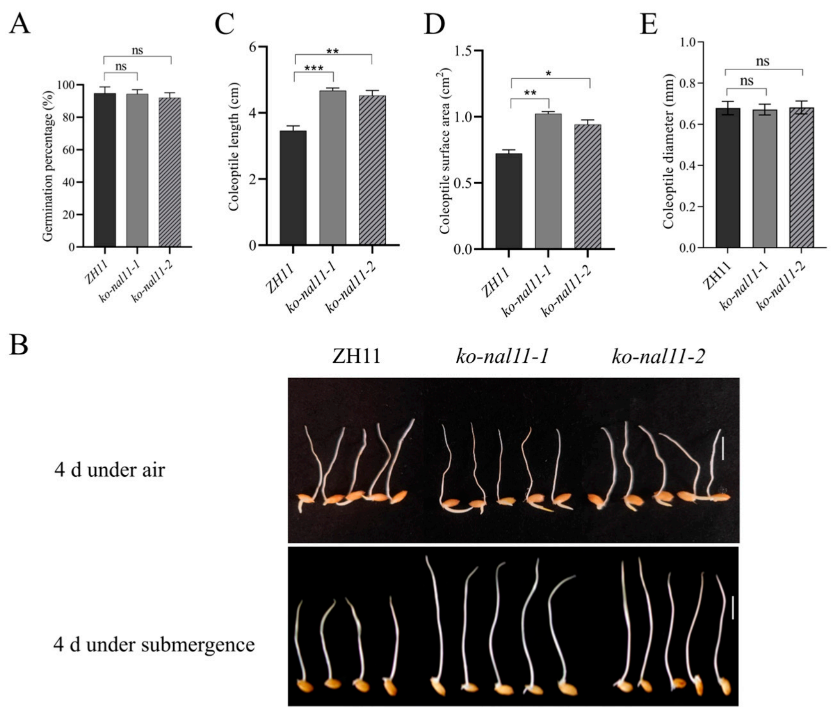

Fig. S4 *NAL11* regulates rice seedling emergence under submergence. A, Germination percentage after 4 d under normal conditions. B, Representative images of coleoptile length for both WT and knockout lines after 4 d under aerobic conditions and 4 d under submergence conditions. Scale bars, 1 cm. C, D, E, Average coleoptile length, coleoptile surface, and diameter of WT and knockout lines after 4 d under submergence, respectively. (Data are presented as mean ± SD, $n = 5$; significant differences were determined by two-tailed Student's t-tests. *$P < 0.05$, **$P < 0.01$, ***$P < 0.001$, ns, no significance).

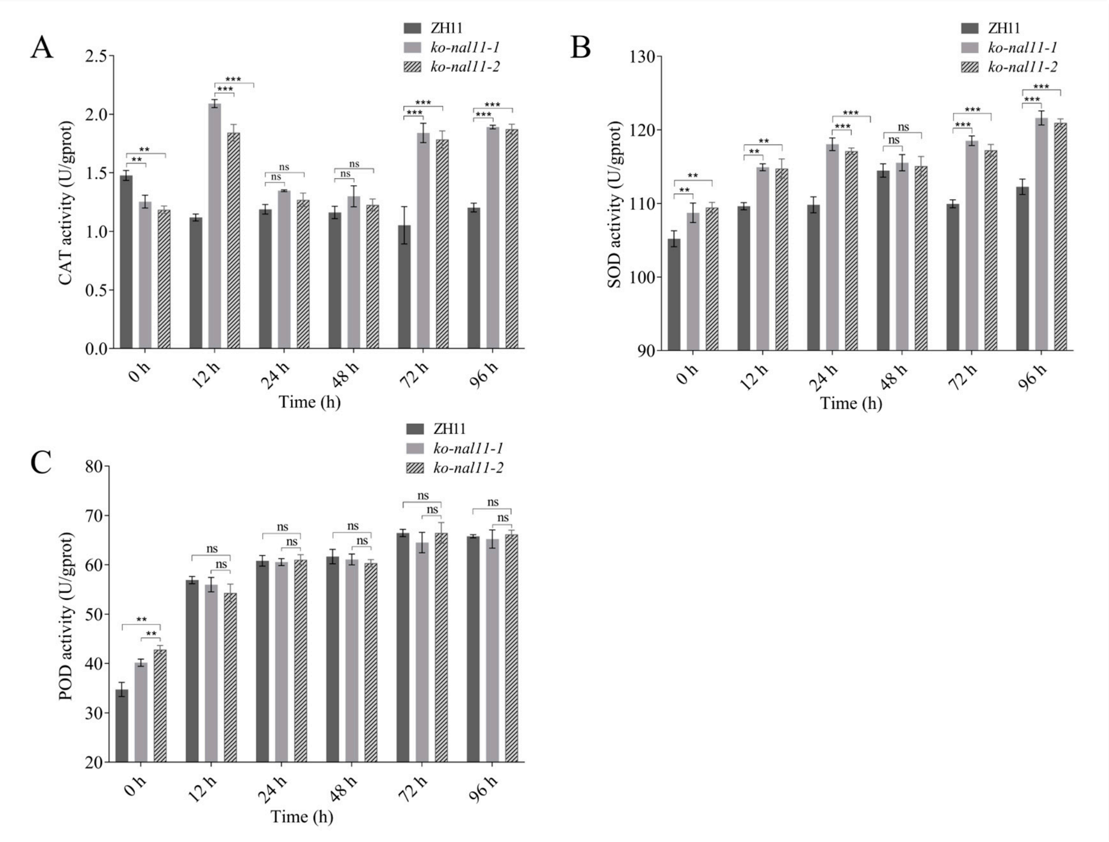

Fig. S5. Analysis of CAT, SOD and POD activity of WT and transgenic plants under normal and submergence conditions. A. CAT activity. B. SOD activity. C. POD activity. (Data are presented as mean ± SD, $n = 5$; significant differences were determined by two-tailed Student's t-tests. **$P < 0.01$, ***$P < 0.001$, ns, no significance).

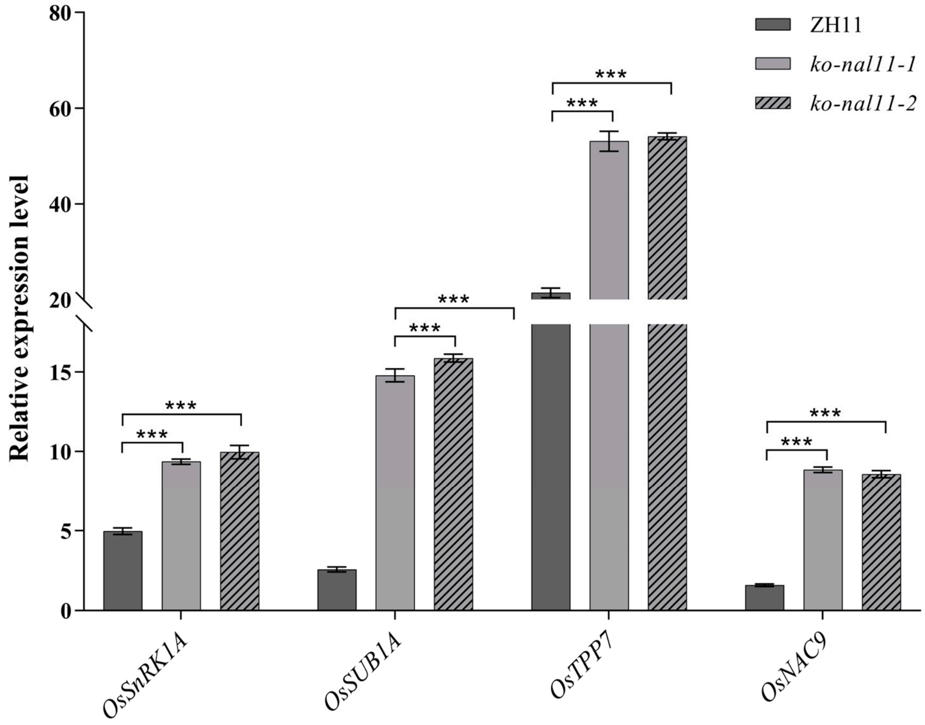

Fig. S6 Transcript accumulation of stress-related genes in seeds after 48 h of submergence treatment. A. *OsSnRK1A*. B. *OsSUB1A*. C. *OsTPP7*. D. *OsNAC9*. (Data are presented as mean ± SD, $n = 3$; Asterisks indicate significant differences between transgenic lines and WT using t-test. ***$P < 0.001$).

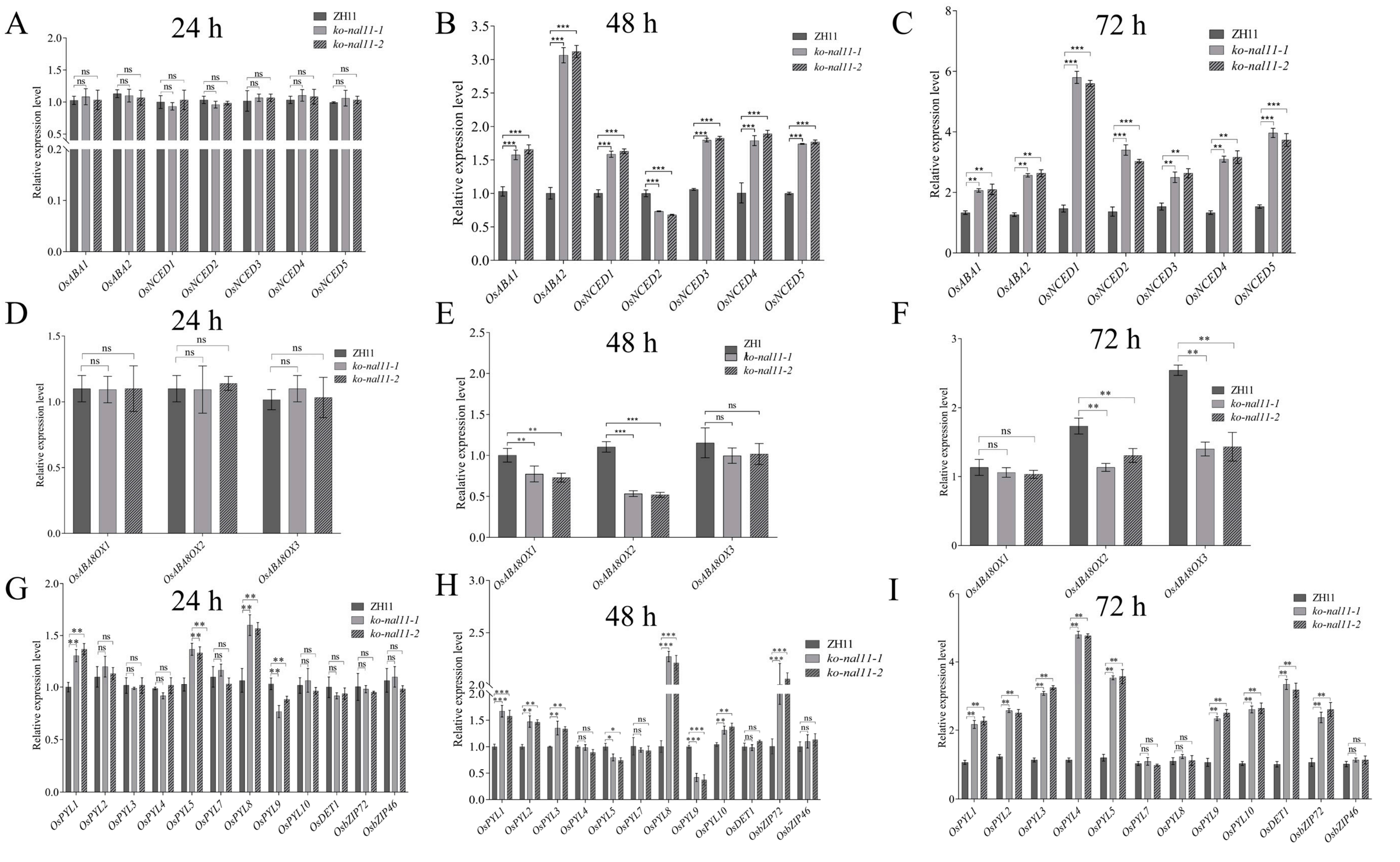
A
24 h
B
48 h
C
72 h
D
24 h
E
48 h
F
72 h
G
24 h
H
48 h
I
72 h
ZH11
ko-nal11-1
ko-nal11-2
Relative expression level
OsABA1
OsABA2
OsNCED1
OsNCED2
OsNCED3
OsNCED4
OsNCED5
OsABA8OX1
OsABA8OX2
OsABA8OX3
OsPYL1
OsPYL2
OsPYL3
OsPYL4
OsPYL5
OsPYL7
OsPYL8
OsPYL9
OsPYL10
OsDET1
OsbZIP72
OsbZIP46
ns
*
**
***

Fig. S7 *NAL11* is involved in the ABA signaling pathway. A-C qRT-RCR of ABA biosynthesis-related genes in the WT and knockout lines at 24 h, 48 h, and 72 h after submergence. D-F qRT-RCR of ABA catabolism-related genes in the WT and knockout lines at 24 h, 48 h, and 72 h after submergence. G-I qRT-RCR of ABA signal transduction-related genes in the WT and knockout lines at 24 h, 48 h, and 72 h after submergence. (Data are presented as mean ± SD, $n = 5$; significant differences were determined by two-tailed Student's t-tests. *$P < 0.05$, **$P < 0.01$, ***$P < 0.001$, ns, no significance).

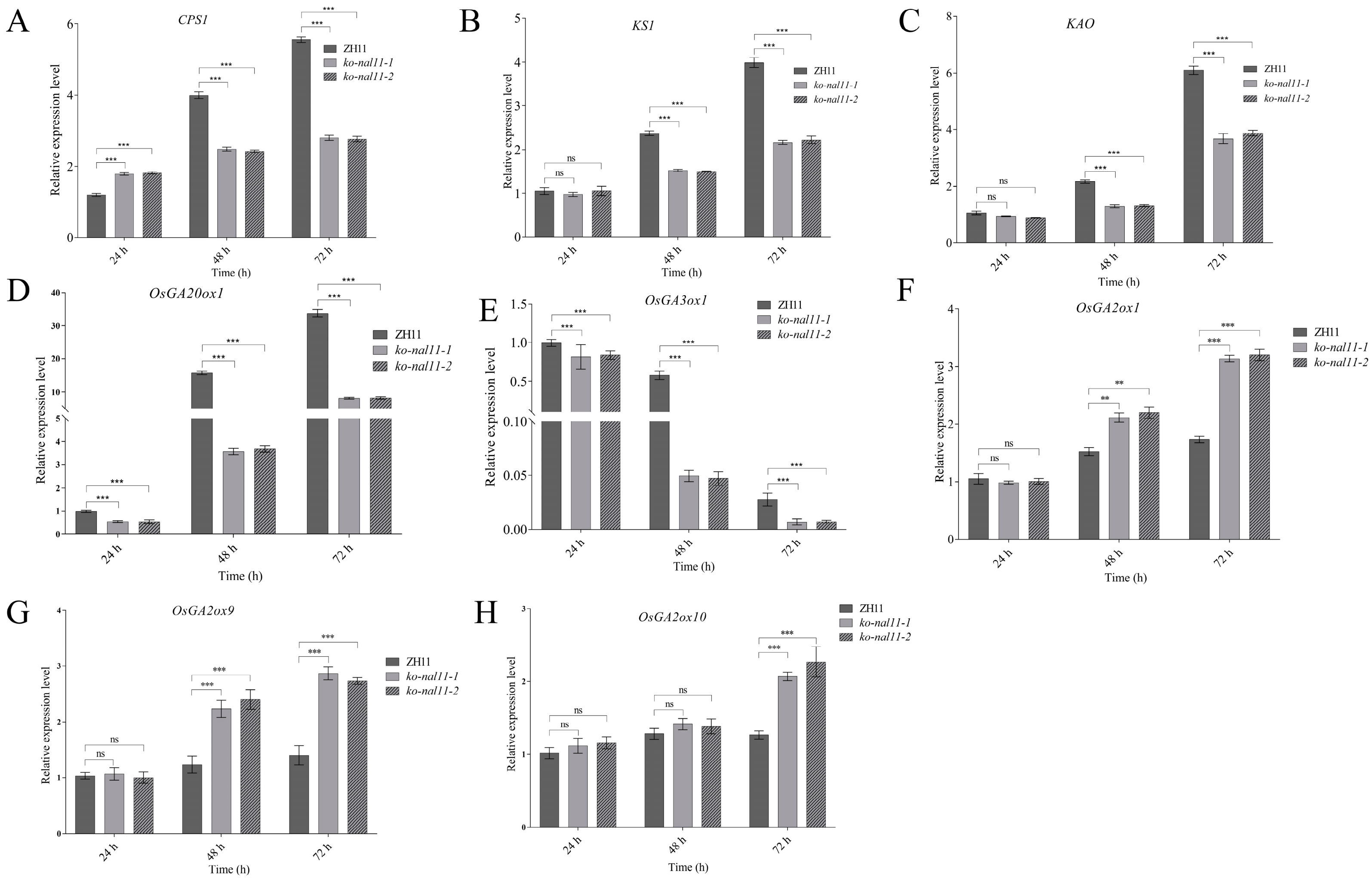

A
CPS1
B
KS1
C
KAO
D
OsGA20ox1
E
OsGA3ox1
F
OsGA2ox1
G
OsGA2ox9
H
OsGA2ox10
Relative expression level
Time (h)
24 h
48 h
72 h
ZH11
ko-nal11-1
ko-nal11-2
***
**
ns

Fig. S8 *NAL11* is involved in the GA signaling pathway. A-E qRT-RCR of GA biosynthesis-related genes in the WT and knockout lines at 24 h, 48 h, and 72 h after submergence. F-H qRT-RCR of GA catabolism-related genes in the WT and knockout lines at 24 h, 48 h, and 72 h after submergence. (Data are presented as mean ± SD, $n = 5$; significant differences were determined by two-tailed Student's t-tests. *$P < 0.05$, **$P < 0.01$, ***$P < 0.001$, ns, no significance).

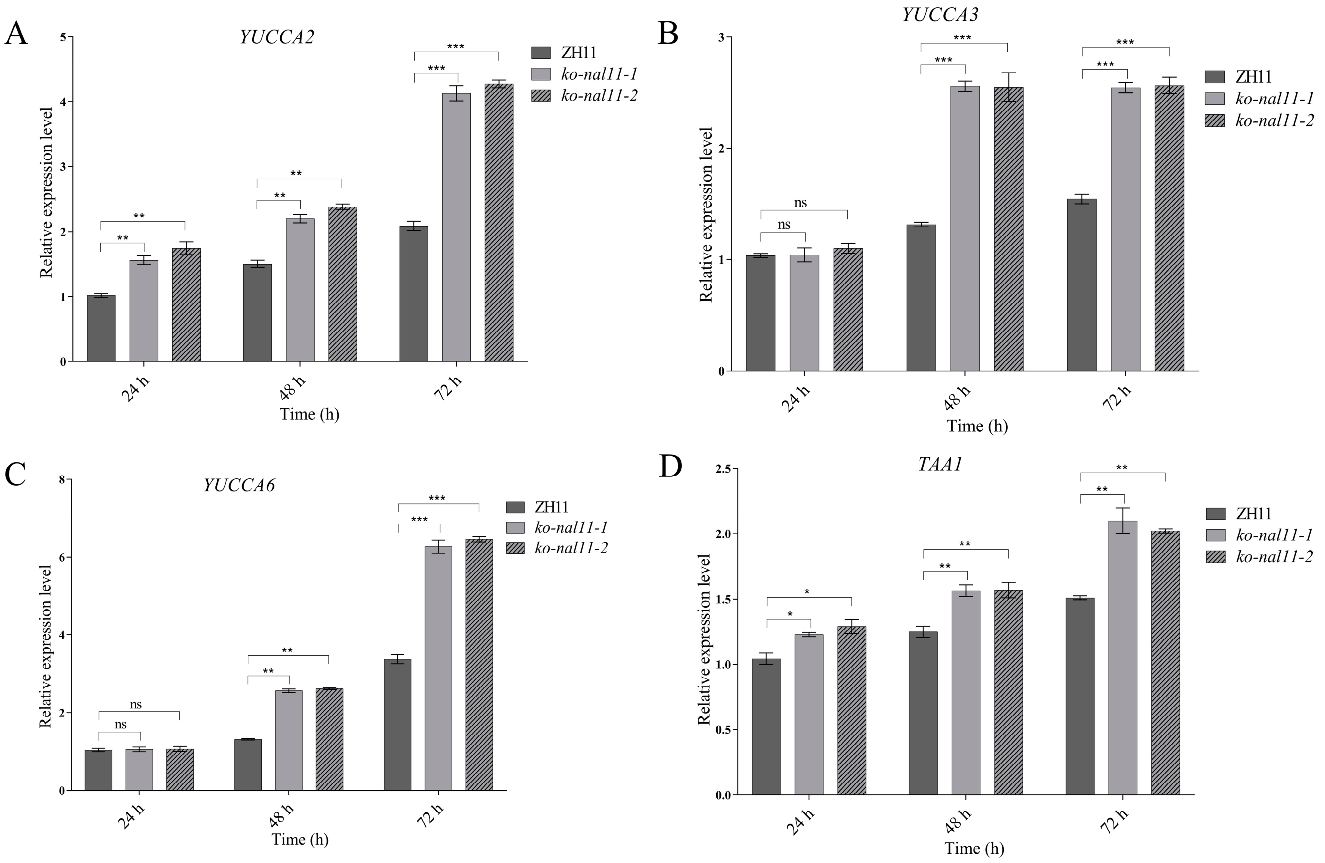

Fig. S9 *NAL11* is involved in the auxin signaling pathway. A-E qRT-RCR of auxin biosynthesis-related genes in the WT and knockout lines at 24 h, 48 h, and 72 h after submergence. (Data are presented as mean ± SD, $n = 5$; significant differences were determined by two-tailed Student's t-tests. *$P < 0.05$, **$P < 0.01$, ***$P < 0.001$, ns, no significance).
