# Supplementary material for: Enhancing Coleoptile Length of Rice Seeds under Submergence through NAL11 Knockout
Source: Plants (Basel). 2024 Sep 17;13(18):2593. doi: 10.3390/plants13182593 (PMC11434697; doi:10.3390/plants13182593)
Supplement: Supplementary file 1 [file plants-13-02593-s001.zip › Supplementary Table S1.pdf]

**A. Plasmid constructions**		
**Primer name**	**Forward primer (5' to 3')**	**Reverse primer (5' to 3')**
NAL11-ts1	taGGTCTCC GGCGGCCCTTGC gttttagagctagaa	cgGGTCTCA CGCCGCTGCAAC tgcaccagccgggaa
NAL11-ts2	taggtctcctcacggttgcaggttttagagctagaa	cgggtctcagtgagtcctgcttgcaccagccgggaa
L5AD5	cgGGTCTCAGGCAGGATGGGCAGTCTGGG CAACAAAGCACCAGTGG	taGGTCTCCAAACGGATGAGCGACAGC AAACAAAAAAAAAAG CACCGACTCG
S5AD5	CGGGTCTCAGGCAGGATGGGCAGTCTG	TAGGTCTCCAAACGGATGAGCGACAG CAAAC
NAL11-GUS	gagctcggtacccggggatccgaggtccgcaatgcagtacaa	ttaccctcagatctaccatggttccggtactccgccacc
**B. Transgenic plants detection**		
**Primer name**	**Forward primer (5' to 3')**	**Reverse primer (5' to 3')**
HPT	atttgtgtacgcccgacagt	gtgcttgacattggggagtt
NAL11-Cas9	tgcatcctaccagtttgtgct	tactgcatcggccttacagtc
**C. RT-qPCR**		
**Primer name**	**Forward primer (5' to 3')**	**Reverse primer (5' to 3')**
NAL11 qPCR	CTACACCACTCATAGCAGGACTCAC	GAAAGCCACCTTCATAGAATTTGCG
OsABA8ox1 qPCR	AAGCTGGCAAAACCAACATC	CCGTGCTAATACGGAATCCA
OsABA8ox2 qPCR	CTACTGCTGATGGTGGCTGA	CCCATGGCCTTTGCT TTAT
OsABA8ox3 qPCR	AGTACAGCCCATTCCCTGTG	ACGCCTAATCAAACCATTGC
OsNCED1 qPCR	AGCCTCGGTCTTCCAATTTT	CACCCAACACAAAAGCTACG
OsNCED2 qPCR	TCATTCCAAAACACCTTCCA	TCCGGGGACCTCCTATGTAT
OsNCED3 qPCR	CGCAACAGTAAAAAGAATTAACAGC	TATACACACACGCGGTCGTT
OsNCED4 qPCR	GAATCCGTAATTGGGCTGAA	CTGGAAGAGGTCCACCTGAG
OsNCED5 qPCR	ACATCCGAGCTCCTCGTCGTGAA	CTCCAGCAACTCTAACCAGCA
OsABA1 qPCR	TATGTGACTGATAATGGAAGTG	ATGGCTGACTGAAGTCTCTCGT
OsABA2 qPCR	GGACCTCATTCATACACAGCAACC	GCATAGGGAGATACGCAGTTCAC
RAmy1A qPCR	CGATGACGAGACTCTCAGTTTA	TAATTCGGATCGGATACAGCTC
RAmy2A qPCR	AAAGGTACGTACTTTCGTCACT	AGCCAAGGCAATGTAGTGTATA
RAmy3A qPCR	GATAGGCTGGTATCCCGAGAAG	TCGTAGAAGATGCATGGTACTC
RAmyC qPCR	GTGGCAAAGATCTACATCGAC	CGTTCTGGTCATAGTCCGG
RAmy3C qPCR	CTGGAGTACCCTGCATTTTCTA	GATCAAGTTGCCCACATCATAC
RAmy3D qPCR	GAGAAGGTCATGGTGAAGATCG	TGACTATCCCGTTCAGTTTAGG
RAmy3E qPCR	GACATCTACGTCGCCATGATC	AGCTAATAGTGCTTTCTACCGG
OsCIPK15	GAGGTGATTAGCAAGATAGGCT	CCTGAATTCACCGTGTTGTATC
OsMYBS1	ATTTGTTCTTTGTGTAGTCGCC	GTGATGATCACTTGCAAACAGT

OsAdh1 qPCR	ACGAGTTTCAGTTCGTCACCCTC	AACCACAACTCGAGCGCACAAATC
OsPDC1 qPCR	GAGATCATCGTGCACAAAGATG	AAGCAGCACATCTTAGACATTG
OsExP2 qPCR	CCATCAACGGGCACTCCTAC	TTGATCGACACGGACTGCAC
OsExP4 qPCR	ATAGTGGAGAAGAGGTTTTGGG	ACTCCCCTTTACTACTACCACT
OsExP7 qPCR	CAGGAAGAAGGGAGGGGTTC	CGAGGACGGAGTTGGACTG
OsExP8 qPCR	TAATCTGGACCGTTCGATGTAG	AAGTCCCAAATTTTCTTCGTGG
OsExP10 qPCR	GCGTGTTTAATTTGTATGCAGC	CACCACCGACATTGCTAATTAG
OsExPB4 qPCR	ACTTAATGGTGGATTCGTGTGA	GAAGTACACAGGGTTCGAGTAC
OsExPB7 qPCR	CTACTCCACTCCTTGCCATTAA	CAACATGCATATACGCACTGAA
OsExPB11 qPCR	AGCCCGTCGCTGTACAAG	CTGGCCGGAGCAAGCT
OsExPB12 qPCR	GCCTTCGAGCCCCATCGAG	TGCAAGGTTGGAACACCGAA
OsExPB15 qPCR	CACCGTTCTCGTCTATGATCG	TATTTCCAGTGCATTTCACCTG
OsPYL1 qPCR	GCCTCACAAATGACGCATATAA	GTAAGAATGAGACAGACAGGGG
OsPYL2 qPCR	CGGTACAAGCACTTCATCAAGT	AGGTGACGGAGCGGTAGTT
OsPYL3 qPCR	CTTTTCAAGCCATTTGTGAGCC	GCAGGCCAGACTTAACATTAAC
OsPYL4 qPCR	CTCAAGAACTACCTCTCGGTC	TCGACGACGTAGGACTCCA
OsPYL5 qPCR	CCTCAGGAACTACTCATCCATC	CGTCCACCACAAATGATTCAAT
OsPYL7 qPCR	ACATCCTCCGTGTCAAGTTTAT	GTTACACCTGAGCAAGTTCTTG
OsPYL8 qPCR	CCTTTTGTGAGAAAGTGCGTAA	TCAAGCAACTCTAACCTCTCAG
OsPYL9 qPCR	GGTGTCGTGTTATTTGTCACTT	CAAACCAAAACAGGCTTCCTTA
OsPYL10 qPCR	CCACTTTCTCTACTCCTAGCAT	GAAGTGCTTGTACACCTGGG
OsDET1 qPCR	CAAACAACAAGAGAGAGCAGAC	CTAACAGTGTTAACAGTAGCGC
OsbZIP72 qPCR	CAGTTTGATTCTGGCGATAAGG	CTCTCCACCACTTTCTCTACTC
OsbZIP46 qPCR	CTTCAGAGTTGCTTCTCTCCTT	GAACTGTAAACGGGATGACATG
OsSnRK1A qPCR	TATTTCCTGGAGTGTGTAGACG	AAAACAGAGAAAGGTTTGCCTC
OsSUB1A qPCR	GACAAACTTCCCACCCGC	GCGCTTCTCCGATTGTGATG
OsTPP7 qPCR	AAGGTGTTCGAGTTCGTGAAG	GAAGAGGACTCCCTTGTTGTG
OsNAC9 qPCR	AACGTGTGTAAATATACAGCGC	TGAAAAACTAAAGAATGCCCCG
CPS1 qPCR	ggatggcagatatgacaaaggca	agatgctgcctcctcaattcg
KS1 qPCR	ctcgatcagctgccatttgc	gcgggcttcagacaattcac
KAO qPCR	CTCCTTCGTGTCCTTCCGTC	ACCACAGCTGAACCTTCCAC
GA20OX1 qPCR	TTCTTCCTCTGCCCGGAGAT	CATGTCGGCCCTGTAGTGG
GA3ox1 qPCR	cggcgatctcttccatgtgc	accgaggaagtagggcatgg
GA2ox1 qPCR	acagccgcctagaactcttca	actggtccataggcatcgtctg
GA2ox9 qPCR	AGCACAGGGTGATGACGAAC	CCTTGTAAGGGGAAGGCTCC
GA2ox10 qPCR	tgagtacgttgtggcggtga	agaccagcctaaccaacgca
YUCCA2 qPCR	ACAATGGTTACCCTCGTATTGT	ACAAGCAGTAAAATCCGGTCTA

YUCCA3 qPCR	TGAAGACATACGATCGTCTCAG	CTGCTCATCATATTCTGCACAC
YUCCA6 qPCR	CTCAAGGGAAGTGACTTCTTCA	TTGTGAAGCCAACAGAGTAGAG
TAA1 qPCR	CTTCTGCAACTTCACCAAGGAG	AATCCTCCACATCCTCCCTATC
OsActin	GAATGCTAAGCCAAGAGGAG	AATCACAAGTGAGAACCACAG
